# Different Patterns of Cortical Electrical Activity by Tactile, Acoustic and Visual Stimuli in Infants: An EEG Exploratory Study

**DOI:** 10.3390/diagnostics15233067

**Published:** 2025-12-02

**Authors:** Rocío Llamas-Ramos, Jorge Juan Alvarado-Omenat, Juan Luis Sánchez-González, Ismael Sanz-Esteban, J. Ignacio Serrano, Inés Llamas-Ramos

**Affiliations:** 1 Department of Nursing and Physiotherapy, Universidad de Salamanca, 37007 Salamanca, Spain; 2 Instituto de Investigación Biomédica de Salamanca (IBSAL), 37007 Salamanca, Spain; 3 FisioSport Salamanca, 37008 Salamanca, Spain; 4 Neurosciences and Physical Therapy Research Group, Department of Physiotherapy, Faculty of Sport Sciences, Universidad Europea de Madrid, 28023 Madrid, Spain; 5 Computational Modeling of Intelligence (COMODIN) Group, Center for Automation and Robotics, CSIC-UPM. Ctra. Campo Real km 0.200, Arganda del Rey, 28500 Madrid, Spain; 6 University Hospital of Salamanca, 37007 Salamanca, Spain

**Keywords:** babies, EEG, stimuli, cortical electrical activity

## Abstract

**Background and Clinical Significance:** Understanding early brain development in infants is essential as identifying an abnormal pattern could accelerate the start of early interventions. There is still limited evidence on how external stimuli (such as tactile, auditory, and visual inputs) influence cortical electrical activity, underscoring the need for integrative studies comparing these modalities in the first months of life. The objective of this paper is to determine the effects of different stimuli (tactile, auditory, and visual) in cortical electrical activity to take advantage of its use in individualized protocols and treatments. **Case Presentation:** An 8-channel electroencephalography cap was placed on the infant’s head to analyze 10 different conditions depending on the selected stimuli: Kangaroo Care with mother and father, rest, tactile stimuli, visual stimuli, acoustic stimuli, and sleep for 5 min. The environment was the same in all conditions to ensure comparison. All conditions have been able to modify the cortical electrical activity presenting different patterns of electrical activity. Tactile stimuli (massage) showed increased activity in the left parietal region. Acoustic stimuli showed increased activity in the frontal region. Visual stimuli presented different patterns, but with a higher occurrence of artifacts due to baby’s movement. **Conclusions**: Acoustic stimuli with music increased cortical electrical activity in frontal region, while tactile stimuli increased the left cerebral hemisphere activity. Future studies are needed to support these exploratory results to establish early interventions in pathological conditions.

## 1. Introduction

Knowledge of brain development in the first months of life is essential to understanding how the foundations of cognitive, sensory, and motor functions are formed. During this period, the brain exhibits high plasticity and sensitivity to external stimuli, which can modulate the maturation of neural networks. However, there are still gaps in our understanding of how different types of stimuli influence cortical activity at such early stages. In this context, electroencephalography (EEG) is a non-invasive and highly sensitive tool for recording cortical electrical activity. This offers the possibility of identifying differential response patterns that contribute to clarifying the mechanisms of child neurodevelopment. It is considered the gold standard to detect seizures or epileptic seizures in newborns but also to check during the first months of life of newborns for any lesion or delay in the brain that may persist throughout life [1]. Immature patterns detected with this method can be considered as biological markers of temporary or permanent brain dysfunction. This is why it is postulated as a diagnostic and prognostic method, since under normal conditions this activity usually disappears at 36 or 37 weeks [2]. Some of the patterns of immaturity reported in the literature are large-amplitude ultralow delta activity, temporal theta activity, delta brushes, or rhythmic theta/alpha/beta activity, which is characteristic of preterm infants [3].

Beyond its role in detecting normal maturation patterns, EEG also provides diagnostic-related insights that are crucial for early identification of specific neurological conditions such as hypoxic–ischemic injury, metabolic encephalopathies, or early epileptic syndromes [4,5]. Quantitative and topographic EEG analyses can help differentiate transient developmental immaturity from pathological alterations, enabling earlier intervention and guiding clinical decision-making. Moreover, the correlation between EEG findings and neuroimaging or neurobehavioral assessments reinforces its diagnostic value as a biomarker of both structural and functional integrity in the neonatal brain [6].

The pediatric population, and specifically preterm infants, is one of the most investigated populations in relation to brain development in the presence of hypoxic–ischemic encephalopathies [7] or even hypothermia conditions [8] from the first hours [9], as well as in autism [10] or epilepsy [11], highlighting the importance of long-term monitoring to be able to observe the evolution of brain development [12].

Most studies present brain reactions in relation to visual [13], acoustic [14,15], tactile [16], and even painful [17] stimuli; however, there is a great variety in terms of type and age of the population, protocols performed, and stimuli applied. These stimuli try to stimulate brain activity in addition to observing the changes that are generated at the brain level to try to understand in a more objective way the brain development of newborns [18]. Unfortunately, it is not clear which is the best or most appropriate stimulus to promote normal brain development.

Although previous research has shown that external stimuli such as touch, sound, and vision can elicit measurable brain responses in infants, significant gaps remain in our understanding of these processes. Tactile stimulation, such as skin contact or massage, has been associated with changes in oxygenation and neural synchronization, yet findings remain inconsistent and poorly characterized by age or sex [19]. Auditory stimuli, particularly speech sounds, have been shown to produce early lateralization of brain activity, with sex-related differences in hemispheric activation; however, the interaction of auditory processing with other sensory systems during early infancy is largely unknown [20]. Visual responses to shape and color begin to mature around nine months, though in younger infants these responses are limited, and their integration with tactile and auditory inputs has not been clearly described [21]. Overall, while each sensory modality independently produces brain activation, there is a lack of studies comparing modalities, examining sex-related differences, and combining both electrical and hemodynamic measures to better capture how the infant brain processes external stimulation. Addressing this gap is crucial for developing evidence-based early stimulation protocols and individualized interventions to support neurodevelopment in the first months of life. These studies provide different conditions evaluated almost always in infants with pathology or with different stimuli. There is no article that has been found that evaluates different conditions in the same population, allowing comparison of the different stimuli in the same subjects and performed in the same environmental conditions. The objective of the present investigation is to verify which is the most suitable external stimuli to increase cortical electrical activity and therefore accelerate brain maturation at an early age.

## 2. Case Presentation

### 2.1. Sample

The sample was composed of a non-preterm baby of seven months as a minimum and who did not present any pathology or treatment at birth as an inclusion criterion; any neuropathy or asphyxia during delivery were considered exclusion criteria. Likewise, the mother did not have to present any previous pathology or complication during pregnancy or at the time of delivery.

### 2.2. Ethical Considerations

The study was approved by the Ethics Committee of the University of Salamanca (Date: 7 July 2022, Code: 806). All data were codified following the Data Protection Law and following the standards established by the Declaration of Helsinki. Parents received an information sheet and signed the informed consent.

### 2.3. Variable

The variable analyzed was the cortical electrical activity (power spectrum density—PSD—in the 4 Hz–30 Hz band) generated during the application of various visual, tactile, and auditory stimuli.

Similarly, the mother’s demographic variables were considered, as well as her medical history.

### 2.4. Material

**Figure 1:**
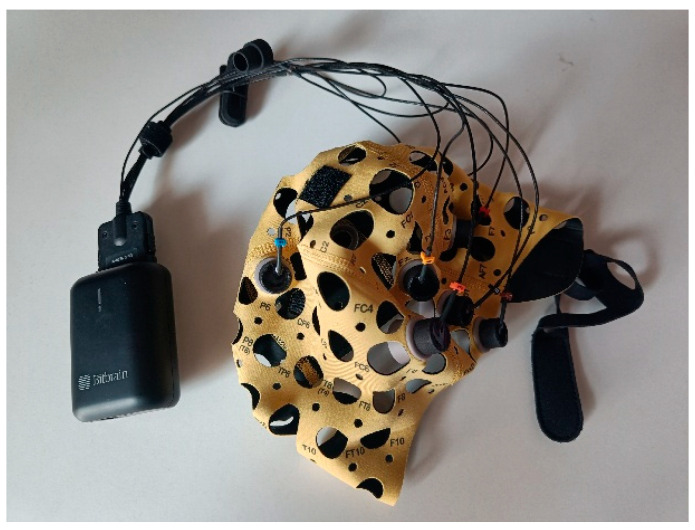
EEG Versatile 8 (Bitbrain).

The EEG Versatile 8 (Bit & Brain Technologies S.L., Zaragoza, Spain) (Figure 1) was selected for the measurement of cortical electrical activity. It consists of a cap and eight electrodes that collect cortical electrical activity signals. The electrode arrangement was established following the “10 system” (Fpz, F3, Fz, F4, P3, P4, O1 and O2) [22], with AFz ground. The activity detected in the infant cap results from postsynaptic potentials of cortical pyramidal cells close to the electrode that receive information from other cells located in other regions with excitatory and inhibitory modifications of glial cells. Each channel represents a voltage potential difference between electrodes, and voltage fluctuation (y-axis) versus time (x-axis) represents EEG waves [23].

### 2.5. Procedure

**Figure 2:**
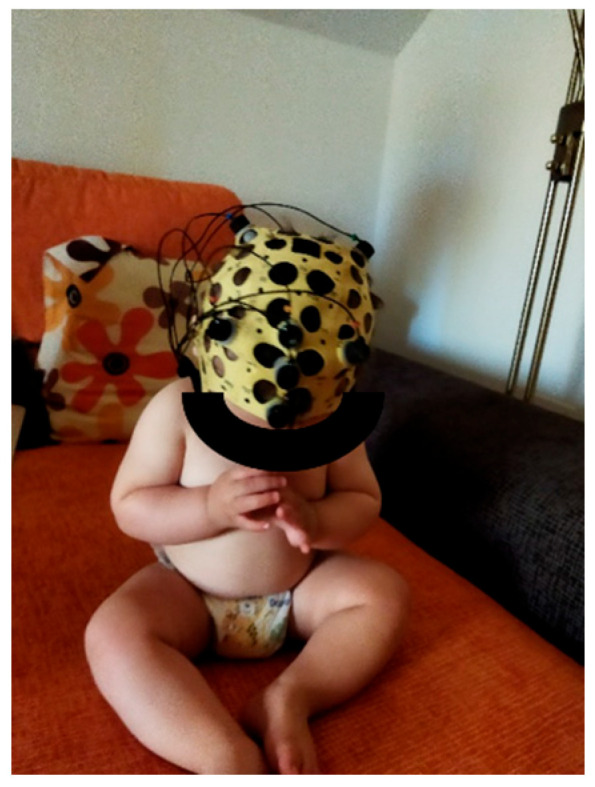
EEG cap.

The study was performed at the infant’s home to ensure peace of mind, in a well-lit room with a pleasant temperature that allowed the infant to be dressed only in a nappy. All conditions were implemented in a random order, on the same day, and in the same room to avoid external confounders. The appropriate cap was selected according to the size of the baby’s head and placed in position (Figure 2). Two physiotherapists participated in the development of the study; one of them oversaw the implementation of each of the conditions and especially of the massage protocol, while the other physiotherapist performed the EEG recording and collected the data. The baby’s parents were present during the whole procedure. A sequence of 10 different stimuli conditions was established to test the differences generated at the level of cortical electrical activity, as well as changes in the electrical activity of the different brain regions.

First, the cap was placed on the baby’s head in the presence of the mother and the physiotherapists (keeping the evaluator who will analyze the data blind). From there, each of the conditions was implemented in random order to avoid preference for some conditions over others. The first condition was a 5 min skin-to-skin recording with the mother, being held in her arms; second, a 5 min resting assessment was performed prior to the third condition, which consisted of soft tactile stimuli without pressure (massage) all over the baby’s body following an established protocol, starting with the lower extremities, upper extremities, and trunk (1 min in each region), after which 5 more minutes of rest were recorded (fourth condition) [24]; fifth, the baby was held in the arms of the father (skin-to-skin). The sixth condition consisted of recording cortical electrical activity while the baby was breastfeeding; the seventh condition was to apply auditory stimuli (favorite baby song) while the baby slept; the eighth consisted of recording cortical electrical activity while the baby slept quietly; the ninth was performed with the same auditory stimuli of the seventh condition but with the baby awake; finally, the tenth situation represented the cortical electrical activity generated while the baby watched and played with his siblings.

The massage was performed by a professional with extensive clinical experience, and the data were analyzed by a researcher skilled in EEG data analysis, blinded to the conditions evaluated.

### 2.6. Data Analysis

EEG signal was recorded at 256 Hz, keeping the impedance of all electrodes under 5 kΩ. The Harvard Automated Processing Pipeline for Electroencephalography (HAPPE v4.1 [25] for developmental data was used to process the EEG, with band-pass filtered (1 Hz–31 Hz) and the rest of the settings of the low-density configured by default, and with data segmented in two-second intervals. The pipeline applies a common average reference to the montage. Artifact rejection is performed by the automated MARA independent component rejection algorithm after wavelet thresholding independent component analysis (W-ICA) and ICA calculations. After processing, electrical noise removal (CleanLine program on 50 Hz), artifact rejection, the number of 2 s intervals, and time duration kept for each condition were as follows: with mother, 110 intervals, 200 s; resting before massage, 110 intervals, 220 s; massage, 133 intervals, 266 s; resting after massage, 118 intervals, 236 s; with father, 122 intervals, 244 s; breastfeeding, 117 intervals, 234 s; auditory stimuli asleep; 122 intervals, 244 s; asleep, 129 intervals, 258 s; auditory stimuli awake, 124 intervals, 248 s; play with siblings, 127 intervals, 254 s. Matlab v24.1.0.2653294 (R2024a) (The MathWorks Inc., Natick, MA, USA) was utilized for preprocessing. Power spectrum density (PSD) (4 Hz–30 Hz) was calculated and plotted with EEGLab v2024.1 [26]. MANOVA analysis was used to test for significant main effects of condition in the PSD of each electrode, after confirmation of data normality with Shapiro–Wilk test and Q-Q plots. We applied Bonferroni correction for multiple comparison for pairwise post hoc analysis. IBM SPSS Statistics v30 (IBM Corp., Armonk, NY, USA) was used for that analysis.

### 2.7. Results

An 8-month-old baby (gestational age: 35 weeks + 5) with no pathology at birth or during the procedure participated in the experiment. All interventions were performed without complications and without harm to the subject on the same day to avoid external causes that could modulate the results. The mother did not present any pathology before, during or after pregnancy, and the delivery was eutocic.

**Figure 3:**
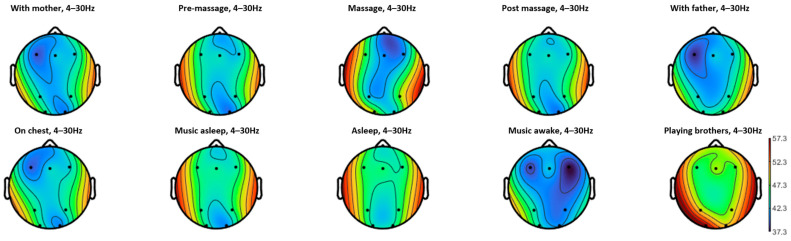
Topographical plots of the PSD between 4 Hz and 30 Hz during each of the ten tasks performed for the subject in the study.

Figure 3 shows the topography of the PSD in the band 4Hz–30Hz during the different tasks for the subject in the study. All channels presented statistically significant main effects of the task on the PSD (Fpz: F(9, 1202) = 7.615, *p* < 0.001, η_p_^2^ = 0.054, 1-β = 1.000; F3: F(9, 1202) = 8.985, *p* < 0.001, η_p_^2^ = 0.063, 1-β = 1.000; Fz: F(9, 1202) = 2.524, *p* = 0.007, η_p_^2^ = 0.019, 1-β = 0.939; F4: F(9, 1202) = 5.974, *p* < 0.001, η_p_^2^ = 0.043, 1-β = 1.000; P3: F(9, 1202) = 8.349, *p* < 0.001, η_p_^2^ = 0.059, 1-β = 1.000; P4: F(9, 1202) = 20.694, *p* < 0.001, η_p_^2^ = 0.134, 1-β = 1.000; O1: F(9, 1202) = 103.478, *p* < 0.001, η_p_^2^ = 0.437, 1-β = 1.000; O2: F(9, 1202) = 7.199, *p* < 0.001, η_p_^2^ = 0.051, 1-β = 1.000), with specially high effect sizes in P4 and O1.

**Figure 4:**
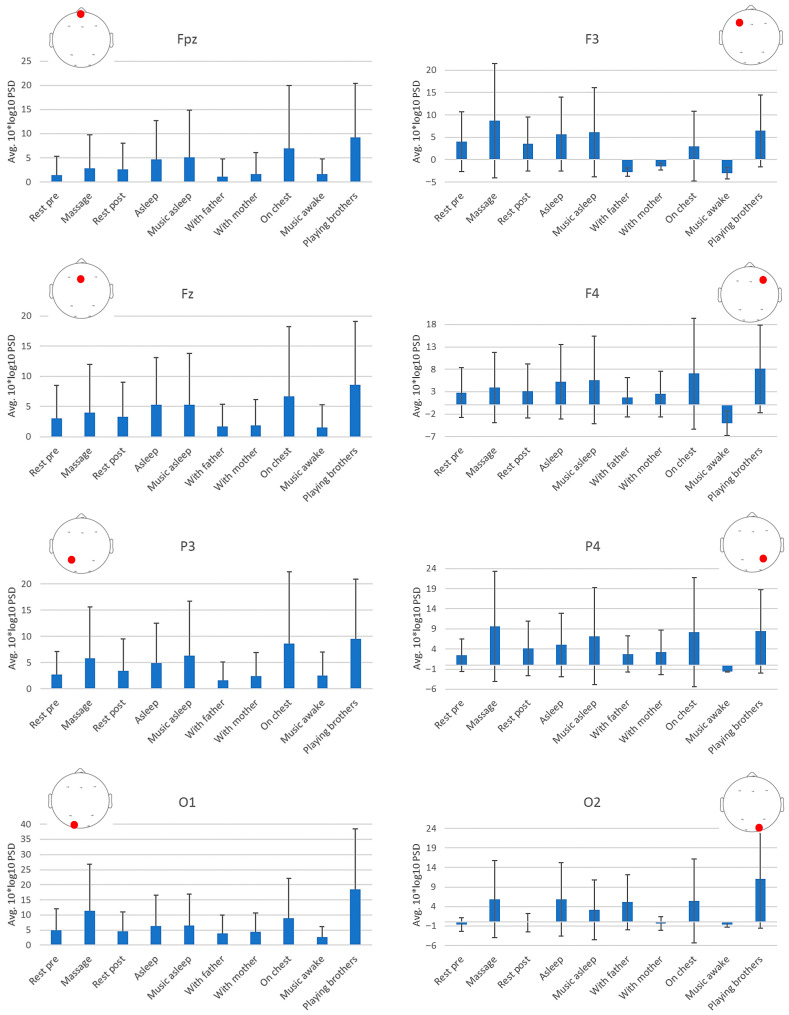
Topographical bar charts of the average and standard deviation (error bars) PSD values for each electrode and task (x-axis).

**Figure 5:**
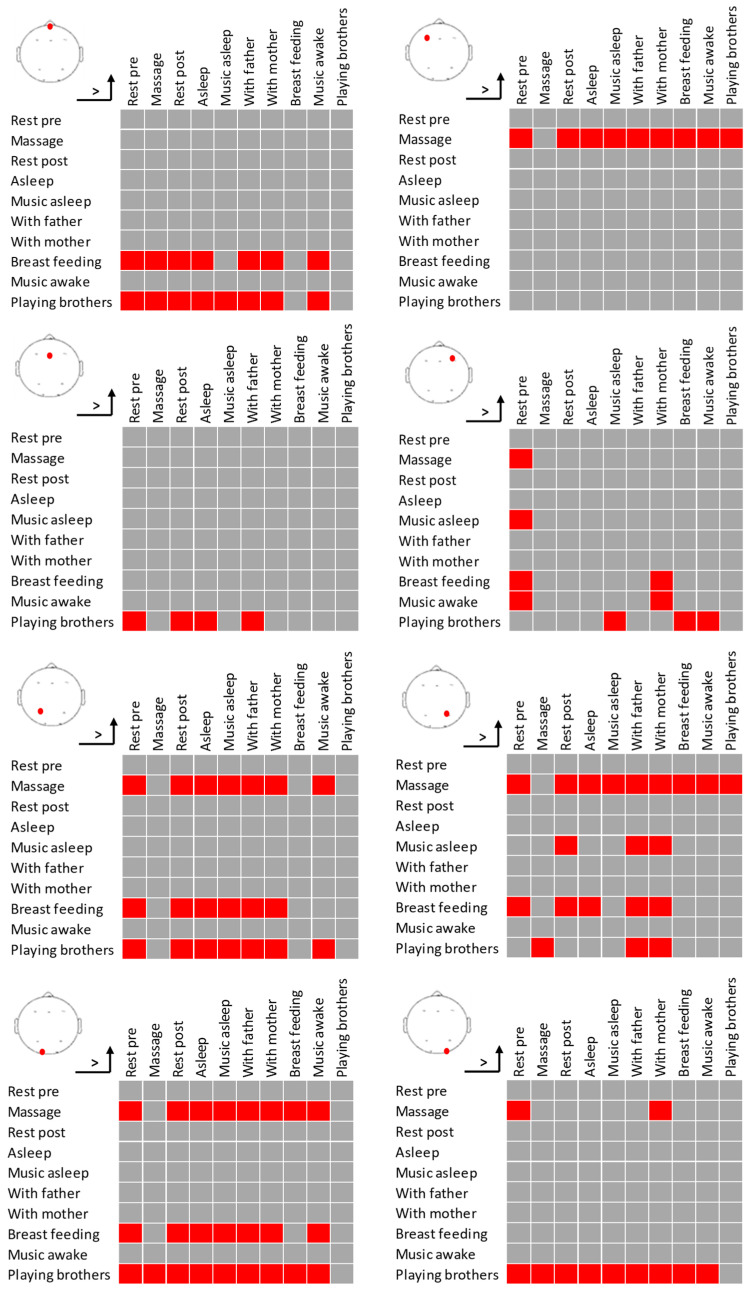
Significant pairwise post hoc differences (Bonferroni-corrected) in PSD for each electrode. Red squares denote that the PSD of the task in the row is significantly higher than the PSD of the task in the column.

The topography results show that the activity distribution is similar when the infant is with the mother, with the father, and on mother’s chest, all showing lower activity in the left frontal area but presenting higher occipital activity with the father. Pre- and post-massage resting intervals show a similar topography, while the massage intervention produces a decreasing activity in the left parietal area but even more reduced activity in the right prefrontal area. Asleep and asleep with music also present similar topographies between them, with slightly higher prefrontal and occipital reduction in PSD during asleep with music, which is also similar to the resting pre- and post-intervention topographies. Finally, listening to music presents an overall decreased PSD but more pronounced activity in the frontal area and particularly lateralized to the right side (Table 1, Figure 4 and Figure 5). This points to music as a highly modulatory stimulus of cortical activity. On the contrary, playing with brothers shows an overall increased activity. However, the number of artifacts during this latter task make this result not reliable.

## 3. Discussion

Several stimuli have shown different patterns of cortical electrical activity in healthy young infants. There is a wide variety of stimuli in the literature that analyze these conditions; however, to our knowledge this is the first study that analyzes stimuli of a different nature in the same subject.

A recent study [22], performed with high-density optical tomography (HD-DOT), shows how social stimuli generate brain responses at the level of the prefrontal area. This agrees with our results, in which the visual stimuli represented the highest modulation of cortical electrical activity when playing with siblings. This must be interpreted with caution because when the infant is left free to play, the recording presents a greater number of artifacts that prevent the correct assessment of the results. This could suggest that there is a processing of social situations in such young infants, which could be useful in the detection of developmental pathologies such as autism or attention deficit hyperactivity disorder by comparing different brain responses to infants with normal development. On the other hand, the data cannot be fully compared since the recording instruments are different, as well as the stimuli and the population; while Collins-Jones et al. [27] used HD-DOT technology, videos of nursery rhymes, and moving toys as stimuli on babies aged 5 to 7 months, in the present study it was performed with EEG, with his siblings as stimuli, and on a baby of eight months.

Regarding acoustic stimuli, studies have shown that in premature babies (born two months before term), there is a cortical structure underlying predictive rhythm processing at the onset of thalamocortical and corticocortical circuits. In our study, these stimuli (the baby’s favorite song) also represented the highest modulation of cortical electrical activity, but we cannot make comparisons due to the difference in age of the sample and the difference in the nature of the stimulus [28].

The tactile stimuli, as well as the rest taken by the mother, present practically the same results as with the father and breastfeeding. In this line of research, another study [29] confirmed that Kangaroo Care, a skin-to-skin, chest-to-chest method of caring for a baby, has effects on neurophysiological activity and brain development in newborns. This practice helps to simulate the womb environment, promotes emotional regulation, and reduces stress [29]. This calming effect is beneficial for the development of infants not only born at term but also in the case of preterm infants, as it reduces cortisol reactivity and increases oxytocin levels, also generating a positive effect on the left hemisphere [30]. Again, these findings agree with our results evidencing increased activity in the left parietal region with tactile stimuli, measured in the case of Hardin et al. [30] with EEG as in the present study.

In addition, a study has been found that combines both auditory and tactile stimuli with infants who heard one pseudoword both whilst being tickled and not tickled. The results showed enhanced ERPs and higher beta-band activity within the left temporal regions, indicating neural processing of acoustic–tactile integration. The importance lies in that this plastic change in neural processing can promote social interaction and effective learning in childhood [20]. In our study, acoustic stimuli have represented one of the stimuli with more activation; however, we do not combine stimuli (which could lead to an accumulation of effects), preventing us from comparing results.

Finally, sleep is another of the most evaluated conditions in this population. Specifically, sleep together with acoustic stimuli has been the subject of a study by Laguna et al. [31], who confirmed in their studies that acoustic stimuli during sleep have an influence on cortical electrical activity in infants. These stimuli perceived during sleep seem to activate specific patterns in brain waves, specifically in the theta and delta bands, which are associated with sleep regulation but also with memory consolidation (in adults and perhaps also in children). The importance of this finding is particularly relevant in the case of newborns since their brain is more vulnerable and still developing. These acoustic stimuli could have an influence on cognitive development but also on long-term emotional development. In addition, Anderson et al. [32] at Oxford University have conducted research on infants. They used controlled sound stimuli while the infants were asleep to test how the brain reacts to them and consolidates these auditory experiences to see how early neural plasticity can be enhanced. In this way, they concluded that exposure to certain sounds during sleeping hours could have benefits in learning and perhaps in early brain development in newborns, presenting an effect at the neurocognitive level.

Most of the localized studies have been performed with MRI; however, EEG has proven useful in monitoring cortical electrical activity in this population. This method of evaluation allows the analysis of specific patterns such as seizures (sometimes unnoticed in the clinical setting), laying the groundwork for diagnosis and even early and individualized interventions to protect, monitor, and watch over the evolution and brain development of newborns [33,34]. In the present study, EEG was used given its easy implementation and the absence of discomfort for newborns, being a safe and noninvasive technique.

Some conditions presented high standard deviations in some channels (such as massage in F3, for example). Those conditions, concretely, the playing with brothers and the breastfeeding conditions, given their nature, might still contain movement artifacts not removed in the preprocessing of the signal, contributing to such a high standard deviation. Other high standard deviations in other conditions and channels are likely due to the stimuli variations perceived during the task interval, such as the massage and auditory conditions.

All possible environmental factors were controlled, primarily lighting and temperature, so that the baby could be comfortable wearing only a nappy. The only people present during the entire process were the physiotherapist and the mother, in order to avoid distractions. Only the father and siblings entered the room when the baby’s condition was being assessed. In addition, considering the number of conditions evaluated, it was decided to establish a 5 min evaluation period to avoid fatigue or discomfort for the baby. All conditions were applied randomly according to the baby’s comfort and were performed after sleeping and eating, so that he was rested and receptive to the evaluations. Finally, the greatest control was achieved by performing all the tests on the same day and on the same subject to avoid changes in environmental conditions.

This study has several clinical implications: it highlights the potential for early identification and targeted intervention in neurodevelopmental disorders. Understanding the normal patterns of brain activation elicited by everyday stimuli (such as tactile contact, acoustic exposure to music or speech, play interactions with siblings, or maternal presence) is essential to establish a baseline of typical neurophysiological responses in term infants. In addition, detecting normal and immature EEG patterns reinforces its value as a marker of brain development or dysfunction [35]. This normative knowledge provides a critical reference for identifying deviations in populations at risk, such as preterm or neurologically vulnerable infants. By characterizing how healthy newborns respond to multisensory experiences, researchers can better interpret altered activation patterns in pathological conditions and design evidence-based early interventions aimed at promoting optimal neurodevelopment. Moreover, mapping these normative responses contributes to developing individualized stimulation protocols that may enhance neural plasticity and compensate for early sensory deprivation in at-risk populations [36,37,38]. On the other hand, by detecting atypical neural activation patterns in response to specific sensory inputs, clinicians can identify early markers of conditions such as autism spectrum disorders, sensory processing disorders, or developmental delays, enabling timely and more effective interventions. For instance, diminished EEG responses to tactile stimuli might suggest somatosensory deficits that warrant early therapeutic stimulation. These findings also support the development of personalized sensory-based intervention programs and inform the design of pediatric rehabilitation strategies that integrate multisensory experiences to promote neurological maturation. Additionally, EEG can serve as a valuable tool for longitudinal monitoring, helping clinicians track developmental progress and evaluate the impact of interventions over time. This sensory-focused approach fosters more precise and early clinical decisions, improving outcomes by tailoring care to the infant’s unique neurophysiological profile.

The main limitation of the study is that it is performed on a single subject; however, it should be noted that the strengths of the study are that all situations have been performed on the same subject and in the same environmental conditions. This allows comparisons to be made and avoids potential individual biases that arise when comparing multiple subjects, and the conditions were implemented randomly. The justification for the small sample size lies in the design of an exploratory study, trying to obtain preliminary data to support a study with an adequate sample size calculated from these initial data, thus avoiding disturbing many subjects in this vulnerable population without being certain of its usefulness. In addition, the low number of electrodes can only provide a coarse-grained analysis of the topography, but it is enough to observe different patterns that justify further research with more subjects and electrodes. Moreover, the differences found between conditions involving playing with siblings and breastfeeding must be interpreted carefully. These two conditions implied movement of the subject, and the signal might contain movement artifacts not removed in the preprocessing step that could alter the PSD. In addition, there is no test–retest because the aim is not to verify whether the degree of activation is greater or lesser but rather to verify whether there are changes in cortical electrical activity with different stimuli. Finally, the exploratory nature of this study should be clearly emphasized to manage expectations and guide future research. As an initial investigation, the findings serve as a foundational step rather than conclusive evidence. Highlighting this exploratory framework allows readers and clinicians to interpret the results as preliminary and hypothesis-generating rather than definitive. This perspective encourages cautious interpretation while underscoring the study’s value in identifying potential neural response patterns that merit deeper exploration. It also sets the stage for future studies with larger sample sizes, more diverse populations, and refined methodologies. By acknowledging the study’s exploration scope, researchers can better position their work within the broader context of early neurodevelopmental research and stimulate further investigation aimed at validating and expanding these early insights. The results, although they must be interpreted with caution, lay the groundwork for future research, with promising data related to brain maturation, which is crucial in the case of preterm infants.

## 4. Conclusions

All the stimuli applied have shown changes in cortical electrical activity, with the acoustic stimuli with music being the one that generates the greatest activation in the frontal region, as well as the tactile stimuli generating the greatest activation in the left cerebral hemisphere. Mapping these responses contributes to developing individualized stimulation protocols for vulnerable populations. Future studies with a larger sample size are necessary to support these results.

## Figures and Tables

**Table 1:** Average (standard deviation) values of the normalized (10*log10) PSD for each task (rows) and electrodes (columns). Values not sharing any subscript (a,b,c,d,e,f,g,h) in each column presented significant statistical post hoc difference. (*p* < 0.05, Bonferroni corrected).

	Avg. 10*log10 PSD (std)							
	Fpz	F3	Fz	F4	P3	P4	O1	O2
**Rest pre**	1.367 _a_ (3.904)	4.028 _a_ (6.650)	3.061 _a_ (5.397)	2.782 _e,f_ (5.580)	2.749 _a_ (4.399)	2.475 _a,c,e_ (4.033)	4.982_a_ (7.065)	−0.576_a,c_ (1.722)
**Massage**	2.855 _a_ (6.867)	8.750 _b_ (12.784)	4.006 _a,b_ (7.989)	3.934 _b,c,d,g,h_ (7.882)	5.811 _c,d_ (9.765)	9.641 _d_ (13.648)	11.430 _d_ (15.381)	5.909 _d_ (9.922)
**Rest post**	2.598 _a_ (5.440)	3.544 _a_ (6.021)	3.255 _a_ (5.755)	3.143 _a,e,g_ (6.014)	3.427 _a_ (6.121)	4.129 _c,e_ (6.731)	4.654 _a_ (6.432)	−0.120 _a,d,e_ (2.344)
**Asleep**	4.701 _a_ (8.001)	5.731 _a_ (8.213)	5.269 _a_ (7.841)	5.247 _a,e,g_ (8.311)	4.898 _a_ (7.612)	5.004 _a,c,e_ (7.916)	6.365 _a_ (10.145)	5.834 _a,d,e_ (9.370)
**Music asleep**	5.101 _a,b_ (9.827)	6.180 _a_ (9.971)	5.304 _a,b_ (8.530)	5.617 _a,b,h_ (9.812)	6.298 _a_ (10.462)	7.205 _a,b_ (12.061)	6.588 _a_ (10.351)	3.188 _a,d,e_ (7.645)
**With father**	1.130 _a_ (3.662)	−2.807 _a_ (−0.941)	1.721 _a_ (3.672)	1.748 _a,e,g_ (4.411)	1.644 _a_ (3.534)	2.767 _e_ (4.523)	3.892 _a_ (6.035)	5.167 _a,d,e_ (7.070)
**With mother**	1.664 _a_ (4.368)	−1.493 _a_ (0.766)	1.876 _a,b_ (4.240)	2.481 _e,h_ (5.116)	2.461 _a_ (4.439)	3.208 _e,f_ (5.516)	4.354 _a_ (6.291)	−0.322 _c,e_ (1.766)
**Breastfeeding**	6.914 _b,c_ (13.062)	3.007 _a_ (7.783)	6.687 _a,b_ (11.502)	7.041 _a,d_ (12.371)	8.603 _b,c_ (13.709)	8.183 _b_ (13.602)	9.013 _b_ (13.202)	5.458 _a,d,e_ (10.748)
**Music awake**	1.613 _a_ (3.221)	−2.989 _a_ (−1.293)	1.589 _a,b_ (3.709)	−4.063 _a,c_ (−2.675)	2.589 _a,b_ (4.415)	−1.595 _a,b,c,e_ (−0.128)	2.725 _a_ (3.377)	−0.610 _a,d,e_ (0.663)
**Playing with brothers**	9.229 _c_ (11.217)	6.468 _a_ (8.024)	8.576 _b_ (10.494)	8.083 _e,g_ (9.738)	9.561 _c_ (11.402)	8.421 _a,b,c_ (10.379)	18.460 _c_ (20.052)	11.099 _b_ (12.666)

## Data Availability

The raw data supporting the conclusions of this article will be made available by the corresponding author on request.

## Abbreviations

The following abbreviations are used in this manuscript:

EEG	Electroencephalography
HZ	Hertz
HD-DOT	High-density optical tomography
PSD	Power spectrum density
